# High dietary diversity is associated with obesity in Sri Lankan adults: an evaluation of three dietary scores

**DOI:** 10.1186/1471-2458-13-314

**Published:** 2013-04-08

**Authors:** Ranil Jayawardena, Nuala M Byrne, Mario J Soares, Prasad Katulanda, Bijesh Yadav, Andrew P Hills

**Affiliations:** 1 Institute of Health and Biomedical Innovation, Faculty of Health, Queensland University of Technology, Brisbane, QLD, Australia; 2 Diabetes Research Unit, Faculty of Medicine, University of Colombo, Colombo, Sri Lanka; 3 Curtin Health Innovation Research Institute, School of Public Health, Faculty of Health Sciences, Curtin University, Perth, WA, Australia; 4 Department of Biostatistics, Christian Medical College, Vellore, India; 5 Mater Mother’s Hospital, Mater Medical Research Institute and Griffith Health Institute, Griffith University, Brisbane, QLD, Australia

**Keywords:** Diet diversity, Dietary variety, DDS, Sri Lanka, Obesity, Adults

## Abstract

**Background:**

Dietary diversity is recognized as a key element of a high quality diet. However, diets that offer a greater variety of energy-dense foods could increase food intake and body weight. The aim of this study was to explore association of diet diversity with obesity in Sri Lankan adults.

**Methods:**

Six hundred adults aged > 18 years were randomly selected by using multi-stage stratified sample. Dietary intake assessment was undertaken by a 24 hour dietary recall. Three dietary scores, Dietary Diversity Score (DDS), Dietary Diversity Score with Portions (DDSP) and Food Variety Score (FVS) were calculated. Body mass index (BMI) ≥ 25 kg.m^-2^ is defined as obese and Asian waist circumference cut-offs were used diagnosed abdominal obesity.

**Results:**

Mean of DDS for men and women were 6.23 and 6.50 (p=0.06), while DDSP was 3.26 and 3.17 respectively (p=0.24). FVS values were significantly different between men and women 9.55 and 10.24 (p=0.002). Dietary diversity among Sri Lankan adults was significantly associated with gender, residency, ethnicity, education level but not with diabetes status. As dietary scores increased, the percentage consumption was increased in most of food groups except starches. Obese and abdominal obese adults had the highest DDS compared to non obese groups (p<0.05). With increased dietary diversity the level of BMI, waist circumference and energy consumption was significantly increased in this population.

**Conclusion:**

Our data suggests that dietary diversity is positively associated with several socio-demographic characteristics and obesity among Sri Lankan adults. Although high dietary diversity is widely recommended, public health messages should emphasize to improve dietary diversity in selective food items.

## Background

Dietary diversity and variety have long been recognized as key elements of high quality diets. A diverse diet increases the probability of nutrient adequacy among adults [1] and leads for positive health outcomes such as reduced complications of diabetes [2], incidence of several cancers [3,4] and all-cause mortality [5]. As dietary factors are associated with increased risk of chronic diseases, local and international dietary recommendations promote increased dietary diversity but limiting saturate fats, refined sugar and salt. However, lack of dietary diversity is a major nutritional concern over among deprived people from the low income countries [6]. Changing from a monotonous diet to one with varied food types has been shown to improve energy and nutrient intakes in the people from developing countries. The demographic and economic transition that many developing countries are undergoing is producing important changes in diet and lifestyle that greatly impact on disease risks [7]. Despite undernutrition and nutrient deficiencies were major concern in the developing countries, recent nutritional transition and changes in the physical activity patters, diet related metabolic problems has emerged as an alarming public health problem in many developing countries particularly among urban dwellers [8].

Sri Lanka is a low-middle income country with undergoing rapid epidemiological and nutritional transition. Despite nutritional deficiencies such as iron deficiency anemia, vitamin A deficiency and protein energy malnutrition are reported in some segment of the Sri Lankan population [9], Non-Communicable Diseases (NCDs) are also emerging as the major diet associated health problem in Sri Lanka. The prevalence of overweight, obesity and central obesity among Sri Lankan adults were 25.2%, 9.2% and 26.2%, respectively in 2005–2006, as defined by Asian Body Mass Index (BMI) cutoffs [10] and there is a clear upward trend [11]. The age-adjusted prevalence of Metabolic Syndrome among Sri Lankan adults was 24.3% (95% CI: 23.0–25.6) [12]. The prevalence of obesity related metabolic problems such as diabetes and hypertention among Sri Lankan adults were 13–14% and 18–19% respectively [13]. Moreover, in Sri Lanka, diet-related chronic diseases currently account for 18.3% of all deaths and 16.7% of hospital expenditure [14].

Although prevalence rates are higher in affluent countries, obesity and abdominal obesity are becoming a major public health concerns in South Asia [15]. Causes for obesity is multi-factorial, among them various dietary factors play an important role. Association between individual nutrients and obesity/abdominal obesity has widely researched, but there is little attention has given on overall dietary diversity and obesity/abdominal obesity. Dietary diversity is an indicator of overall diet. Higher diet variety is associated with increased intake of fiber and vitamins [16] and on the other hand, increased variety contributes to high calorie consumption [17]. Sri Lankan has interesting socio-economical relationship with obesity. For instance, higher wealth and education is positively associated with obesity among Sri Lankan adults [10]. Therefore, evaluating the association between diet diversity and obesity would be interesting in this population. Explore the underline associations between obesity and dietary diversity is very important as lifestyle intervention can change dietary diversity in deferent populations. The aim of this study was to explore association of diet diversity with obesity in Sri Lankan adults.

## Methods

### Study design and sample selection

Subjects were recruited from a subset of Sri Lanka Diabetes and Cardiovascular Study [18]. Six hundred adults aged > 18 years were randomly selected by using multi-stage stratified sample. The details of sample selection have been published elsewhere [10,19]. Ethical Approval for this study was obtained from the Ethical Review Committee, Faculty of Medicine, University of Colombo, Sri Lanka and informed consent was obtained from the subjects before the data was collected.

### Dietary assessment

Dietary intake assessment was undertaken by a 24 hour dietary recall by trained nutritionists in a random day to obtain the ‘usual’ intake. Although multiple 24 hours may represent the ‘usual’ intake better, a single 24-hour recall is considered as the best reference period to assess dietary diversity and longer reference periods result in less accurate information due to imperfect recall [20]. However, if previous 24 hour period is atypical due to special occasion or illness, a different day was selected for the interview. We collected a detailed description of the foods eaten and amount was estimated by using food photographs and common household utensils. For mixed dishes, food types were disaggregated before ingredients were categorized into appropriate food groups as detailed earlier [19].

### Socio-demographic and anthropometric

Socio-demographic details and clinical status (self-reported diabetes) were collected from interviewer administrated questionnaire. Height was measured using a portable Holtain Stadiometer (Chasmors Ltd, London, UK) to the nearest 0.1 cm. Body weight was measured using a SECA electronic scale (Hamburg, Germany) to the nearest 0.1 kg. Most of the participants were weighed wearing light clothes and after fasting. Waist Circumference was measured using a tape to the nearest 0.1 cm at the midpoint between the lower costal border and the top of the iliac crest, at the end of normal expiration. BMI was calculated by dividing body weight (in kilograms) by height (in meters squared). Definition of overweight (BMI ≥23 kg.m^-2^), obesity (BMI ≥25 kg.m^-2^) and abdominal obesity (Men: WC ≥ 90 cm; Women: WC ≥ 80 cm) were categorized according to Asia-pacific anthropometric cut-offs [10].

### Dietary diversity score (DDS)

A DDS was defined as the total count of different food groups irrespective of the amount consumed by individuals over the 24 hour period. All the food items consumed by the subjects were categorized into 12 food groups which were starch (cereals, tubers, roots and starchy vegetables such as jackfruits), vegetables, green leafy vegetables (green salads and ‘*Mallum*’), fruits, fish (including dried fish and seafood) meat (including poultry, egg), legumes (including nuts and seeds except coconut), milk (including all dairy products), beverages (tea, coffee and fizzy drinks), oils and fats (coconut products were included), sweets and miscellaneous (e.g. Alcohol). The choice of the 12 food groups was based on the local and international food grouping techniques adapting cultural context [20,21]. So the maximum score was 12, one point given for each group consumed during the registration period.

### Dietary diversity score with portions (DDSP)

We defined DDSP considering major food groups in the Sri Lankan food pyramid Starch, Vegetables, Green leafy vegetables, Meat [meat/poultry/egg], Fish [fish/dry fish/sea foods], Milk [Milk/dairy products], Pulses and Fruits [21]. DDSP was calculated applying a minimum consumption of one portion for respective food groups. Details of the portion sizes were published previously [19]. The maximum score for DDSP was 8.

### Food variety score (FVS)

FVS was defined as the number of different food items eaten during last 24 hour [16]. The total number of foods included irrespective of quantity consumed. There is no maximum value here.

### Data analysis

Statistical Package for Social Sciences software version 16 (SPSS Inc., Chicago, IL, USA) was used to conduct all the statistical analyses. Descriptive data are presented as means and SDs. Percentage of consumption of different food groups according to DDS and DDSP were sorted. DDS and FVS were further categorized to groups according to DDS and FVS values. Then BMI, WC and energy intake were calculated for the groupings of dietary diversity values. Total energy was analyzed using NutriSurvey 2007 (EBISpro, Germany) software. Independent samples test and ANOVA were used to compare the means. For all statistical tests, a P value < 0.05 was accepted as significant.

## Results

Response rate was 80% (n=481) and details of the subjects’ characteristics were reported in Table 1. Mean of DDS for men and women were 6.23 and 6.50 (p=0.06), while DDSP was 3.26 and 3.17 respectively (p=0.24). FVS values were significantly different between men and women 9.55 and 10.24 (p=0.002). Several socio-demographic parameters were significantly associated with all three dietary diversity parameters. People living in the estate areas had the lowest DDS, DDSP and FVS compared to both urban and rural. Similarly, Indian Tamils had lowest values for all three diet diversity parameters. Higher education level is associated with increased dietary scores but not for age categories. Adults with BMI ≥25.0 kg.m^-2^ had highest DDS, DDSP and FVS values. Centrally obese participants had significantly higher DDS, DDSP and FVS values but no significant difference was seen between diabetic and non-diabetic.

**Table 1:** Mean and SD of dietary diversity score (DDS), dietary diversity score of portions (DDSP) and food variety score (FVS)

**Subjects’ characteristics**		**DDS**			**DDSP**			**FVS**	
	**Mean**	**SD**	**P value**	**Mean**	**SD**	**P value**	**Mean**	**SD**	**P value**
**Gender**									
Male (n= 168)	6.23	1.57	0.06	3.26	1.18	0.24	9.55	2.42	0.002
Female (n= 313)	6.50	1.53		3.17	1.15		10.24	2.22	
**Area of residence**									
Urban (n=157)	6.67	1.53	<0.001	3.47	1.12	0.001	10.55	2.26	<0.001
Rural (n=285)	6.38	1.56		3.14	1.17		9.93	2.31	
Estate (n=39)	5.64	1.27		2.56	0.99		8.28	1.62	
**Ethnicity**									
Sinhalese (n= 373)	6.56	1.58	<0.001	3.22	1.16	0.012	10.18	2.36	<0.001
Muslim (n= 28)	6.57	1.23		3.50	1.29		10.07	2.28	
Sri Lankan Tamil (n= 42)	5.71	1.27		3.33	1.10		9.83	1.78	
Indian Tamil (n = 38)	5.55	1.25		2.61	0.97		8.21	1.58	
**Educational level**									
No schooling (n=30)	5.83	1.42	<0.001	2.63	1.06	0.001	9.00	2.13	<0.001
Up to grade 5 (n= 122)	6.02	1.45		2.95	1.10		9.31	2.04	
Up to O/L ( n=186)	6.53	1.53		3.20	1.10		10.10	2.26	
Up to A/L ( n= 118)	6.64	1.52		3.51	1.25		10.52	2.48	
Graduate (n= 23)	7.09	1.90		3.61	1.12		11.30	1.99	
**Age category (years)**									
18 – 30 (n= 61)	6.00	1.60	0.051	3.20	1.12	0.14	9.69	2.70	0.39
31 – 40 (n= 84)	6.70	1.56		3.20	1.10		10.31	2.50	
41 – 50 (n=121)	6.60	1.57		3.17	1.18		10.07	2.28	
51 – 60 (n=110)	6.33	1.43		3.25	1.17		9.93	2.15	
> 61 (n=104)	6.26	1.53		3.19	1.22		9.91	2.13	
**BMI category**									
≤ 18.5 kgm^-2 ^(n =74)	5.69	1.52	<0.001	2.86	1.01	0.016	8.86	2.07	<0.001
>18.5 - ≤ 22.9kgm^2 ^(n=172)	6.52	1.47		3.20	1.14		9.99	2.20	
>23 - ≤ 24.9 kgm^-2 ^(n=87)	6.34	1.68		3.11	1.20		10.07	2.25	
≥25.0 kgm^-2 ^(n= 146)	6.67	1.46		3.41	1.19		10.55	2.42	
**Abdominal obesity**									
Yes (n=163)	6.69	1.50	0.005	3.37	1.18	0.046	10.61	2.30	<0.001
No (n= 316)	6.27	1.56		3.10	1.14		9.69	2.27	
**Diabetes mellitus**									
Yes ( n = 60)	6.77	1.65	0.23	3.52	1.09	0.16	10.32	2.30	0.67
No (n= 421)	6.36	1.53		3.15	1.17		9.95	2.32	

Table 2 shows the distribution pattern of consumption of foods from different food groups among Sri Lankan adults according to DDS. Minimum DDS was 2 and maximum was 11 out of 12. As DDS increased, the percentage consumption was increased in most of food groups except starch as everybody consumes starch from DDS value of 2. Cereals were the commonest consumed food groups among Sri Lanka with the lowest DDS and DDSP scores. Milk and dairy product intake increases slowly, but gradually with both DDS and DDSP, whereas meat products are consumed by a significant portion of the population only at higher dietary scores (DDS≥ 8; DDSP≥5). Pulses reach more than 50% from DDS value of 3, followed by vegetables, beverages from DDS of 4. However meat/poultry/egg reached ≥ 50% at the DDS of 10. Similar to DDS patterns, DDSP also showed maximum value for starch group but lower values for green leafy vegetables, meat, milk and fruits (Table 3).

**Table 2:** Percent consumption of different food groups by DDS for Sri Lankan adults (n=481)

**DDS**	**1**	**2**	**3**	**4**	**5**	**6**	**7**	**8**	**9**	**10**	**11**
No of adults	0	1	9	42	89	109	115	72	36	6	2
Percentage of adults with DDS	0	0.2	1.9	8.7	18.5	22.7	23.9	15.0	7.5	1.2	0.4
**Food groups**											
Starch	0	100.0	100.0	100.0	100.0	100.0	100.0	100.0	100.0	100.0	100.0
Vegetables	0	0	33.3	54.8	76.4	69.7	85.2	86.1	94.4	100.0	100.0
Green leaves	0	0	0	21.4	12.4	38.5	42.6	52.8	80.6	83.3	100.0
Meat/poultry/egg	0	0	0	2.4	21.3	20.2	24.3	45.8	38.9	66.7	50.0
Fish/dry fish/sea foods	0	0	44.4	33.3	52.8	66.1	70.4	87.5	97.2	100.0	100.0
Milk/diary products	0	0	22.2	19.0	59.6	60.6	66.1	79.2	83.3	100.0	100.0
Pulses	0	100.0	55.6	54.8	59.6	67.9	77.4	81.9	86.1	100.0	100.0
Fruits	0	0	0	14.3	12.4	33.0	45.2	45.8	77.8	66.7	100.0
Fat/oil	0	0	11.1	26.2	22.5	35.8	47.8	52.8	61.1	33.3	100.0
Beverages	0	0	33.3	64.3	60.7	78.0	86.1	90.3	91.7	100.0	50.0
Sweets	0	0	0	2.4	6.7	14.7	33.0	43.1	50.0	83.3	100.0
Miscellaneous	0	0	0	7.1	15.7	15.6	21.7	34.7	38.9	66.7	100.0

**Table 3:** Percent consumption of different food groups by DDSP for Sri Lankan adults (n=481)

**DDSP**	**1**	**2**	**3**	**4**	**5**	**6**	**7**
No of adults	23	122	149	128	44	13	2
Percentage of adults with DDS	4.8	25.4	31.0	26.6	9.1	2.7	0.4
**Food groups**							
Starch	100.0	100.0	100.0	100.0	100.0	100.0	100.0
Vegetables	0.0	30.3	56.4	71.9	86.4	100.0	100.0
Green leafy vegetables	0.0	3.3	16.1	36.7	38.6	69.2	100.0
Meat	0.0	5.7	20.8	26.6	40.9	53.8	100.0
Fish	0.0	28.7	40.9	64.1	75.0	92.3	100.0
Milk	0.0	3.3	6.0	10.9	27.3	30.8	100.0
Pulses	0.0	24.6	42.3	58.6	77.3	69.2	50.0
Fruits	0.0	4.1	17.4	31.2	54.5	84.6	50.0

Mean BMI, WC and energy intakes were gradually increased with DDS, DDSP and FVS categories (Table 4). Participants with 2–5 DDS value had BMI of 22.16 kg.m^-2^ and WC of 77.0 cm and gradually rise up to BMI of 23.82 kg.m^-2^ and WC of 80.04 cm with DDS 8–11 category. Energy consumption also follows the same pattern.

**Table 4:** Mean BMI, Waist circumference and energy intake of the subjects according to DDS, DDSP and FVS

		**BMI (kgm-**^**2**^**)**			**Waist circumference (cm)**			**Energy intake**		
		**Mean**	**SD**	**P values**	**Mean**	**SD**	**P values**	**Mean**	**SD**	**P values**
DDS	2–5 (n=141)	22.16	4.11	0.002	77.00	10.48	0.034	1705	615	<0.001
	6–7 (n=223)	23.38	4.24		79.39	10.50		1792	597	
	8–11 (n=116)	23.82	3.40		80.04	9.37		2004	580	
DDSP	1–2 (n=144)	22.76	3.94	0.027	77.74	9.93	0.009	1652	609	<0.001
	3 (n=149)	22.84	4.29		77.70	10.22		1739	540	
	4 (n=127)	23.24	4.12		79.80	10.88		1985	617	
	5–7(n=59)	24.54	3.34		82.14	9.19		2079	583	
FVS	3–8 (n =121)	21.76	3.97	<0.001	76.16	10.08	0.002	1651	596	<0.001
	9–10 (n= 271)	23.33	4.46		79.19	11.08		1804	633	
	11 (n=68)	23.28	3.35		78.69	9.03		1826	558	
	12–18 (n= 118)	24.14	3.55		81.16	9.46		2003	570	

## Discussion

Sri Lankan adults had relatively low dietary diversity values, in particularly, relatively higher FVS and DDS and lower DDSP value indicates that although people consume several type of food items, the amount of consumption is low for many food groups. In WHO STEP Survey reported that 3% of Sri Lankan consume more than five fruits and vegetable per day [22]. Rathnayake et al. reported much lower mean DDS (4.4) and FVS (8.4) among group of rural elderly people [23]. DDS, DDSP and FVS values for Indian Tamils were remarkably less than Sinhalese and Muslim ethnic groups. It is reported that malnutrition and nutritional deficiencies are highest in the estate sector where most of Indian Tamils are living [9]. Although we have lack of data on nutrient adequacy in this study sample, it can be postulate that low dietary diversity may causes deficiencies among Sri Lankan adults. People with better education may have high profile occupations and greater purchasing power could lead for higher consumption of different food variety. Although no previous data available on dietary diversity among Sri Lankan adults, children who lived in estate sector had a lower dietary diversity [24]. Moreover, lower maternal education was negatively associated with receiving a diverse diet for those children [24]. Obese and abdominal obese participants had higher DDS, DDSP and FVS values compared to non-obese and non-abdominally obese groups.

Sri Lankans consume excess amount of starchy foods but per below amount of fruits, vegetables and dairy products [19]. Table 2 and 3 showed that 100% values for starch group in every DDS and DDSP indicating starchy staple food is the inclusive food group in Sri Lankan meals. Nearly five percent participants had almost starch meal without significant amount of other food groups for whole day, this is due to some people consume cereal (e.g. rice) with a starchy vegetable (e.g. potato curry). Predominantly carbohydrate diets raise plasma glucose, insulin, triglycérides and non-esterified fatty acids leading to insulin resistance [25]. Total carbohydrate intake is associated with risk of diabetes among South Indian adults [26]. High prevalence of diabetes and its complications among Sri Lankan adults may be associated with starch based but poor variety meals [18,27]. Amongst the food groups of dietary pyramid, meat, green leafy vegetables, milk and fruits were least frequent. Increased intake of fruits and vegetables could play a protective role against obesity associated metabolic risk factors in South Indians who is prone to get premature coronary artery disease [28]. Reasons for monotonous diet among Sri Lankan adults needed to be explored although it is associated with low obesity level. Public Health initiatives to improve appropriate diversity of diet are important.

Torheim et al. reported a positive correlation between energy intake and DDS, as well as variety of different food groups in Mali [29]. When a diet is composed of foods that differ on sensory characteristics such as color flavor and shape may cause hyperphagia [30]. Animal and human studies showed that food intake increases when there is more variety in a meal or diet and that greater dietary variety is associated with increased body weight and subsequently obesity [30]. Dietary variety within food groups was positively associated with body fatness among healthy adults [31]. Several studies showed a positive correlation between calorie intake and dietary diversity [17,29]. On the contrary, an inverse association between DDS and obesity/abdominal adiposity was reported among the female students of Isfahan University [32]. In US women, low BMI was associated with higher DDS; in US men, there was no clear relation of BMI to dietary diversity [5]. Our result shows that positive association between all three dietary diversity indices with calorie intake suggesting that consumption of large number of food items may lead to excessive intake of calorie and weight gain. Although many dietary guidelines promote consumption of varied diet, it should be selective (e.g. vegetables) rather than absolute number. Moreover, Food Guide pyramids are not designed to maintain energy balance, but show nutritional adequacy and balance [17]. Increased dietary diversity in health promotion may not be appropriate for combating obesity epidemic in Sri Lankan. A reduction in dietary variety of highly palatable and energy rich foods may be an appropriate strategy to prevent and treat obesity in Sri Lankan. In the same time, to prevent deficiencies foods with high nutrient but low calories (e.g. green vegetables, low fat milk) should be encouraged.

### Limitations

We used a single 24 hour recall to obtain usual intake, however multiple dietary recalls during weekdays and weekends may provide a better picture of the habitual diet. In the nutritional literature, DDS and FVS are widely used and showed appropriate correlation with nutritional status and association with chronic disease. Simple counting of food groups or consumed food items are used for define diet diversity scores. These methods have limitations. First, although it is not easy to distinguish healthy and unhealthy food items, these counting systems are assigned equal values for every food item respective of the health outcome (e.g. fruits and sweets). Second, the distributions of individual food quantities are not considered by count measures. In this exercise we defined DDSP with consumption of minimum of one portion of respective food groups; however we did not define an upper limit. Drescher et al. recommend considering, health values of a consumed food items for measure healthy food diversity [33]. Another limitation is that we did not measure the diversity within food groups and most of Sri Lankan dishes are mixed in nature, which causes considerable practical limitations for food item groupings. Furthermore, there is no an updated food composition tables for Sri Lankan mixed dishes in particularly for micronutrients. Therefore we were not able to calculate nutrient adequacy ratio and mean adequacy ratio. Moreover, lack of physical activity data among this population limit us to calculate daily energy requirements. Although the main aim of this study is to explore the associations between dietary diversity/variety and obesity.

A follow up study showed an inverse dietary diversity-mortality association was adjusted for potential dietary and socio-demographic confounders [5]. DDS is population specific and there is no standard scoring method, therefore it is invariably difficult to compare DDS values among different countries. Because of cross sectional nature of this study, we cannot express long term health outcome with regards to dietary diversity among Sri Lankan adults. Moreover, obesity is associated with multiple socio-economical factors which could be possible confounding factors for diet diversity. Prospective studies are needed to explore association between dietary diversity and weight gain/obesity.

Acknowledging the limitations of this study, these results showed the dietary diversity, portions consumption and number of food item intakes according to different socio-demographic characteristics. Globally, normative data on ‘ideal’ or ‘target’ levels of diversity are usually not available. Therefore, our results can be used to assess current picture of the dietary diversity among Sri Lankan adults. Repeating similar study in a given time may help to assess improvements in food security and expected changes in the population. Illangasekara et al. reported temporal trends in the prevalence of diabetes mellitus in a rural community in Sri Lanka which is closely accompanied by an increase in the monthly income. But there is a lack of data on changes in dietary habits in this population with related to epidemic of diabetes and other diet associated metabolic disorders. Although our aim was to assess the individual diet diversity, as our sample included predominantly housewives and in Sri Lanka majority of people consume homemade foods. These results can be used as household dietary diversity values. Therefore, these values may indicate socio-economic level of the household [20].

## Conclusion

In conclusion, our data suggests that dietary diversity and variety are associated with obesity among Sri Lankan adults. High dietary diversity is widely recommended as it can be used as proxy indicators of nutrient adequacy. Therefore public health messages should emphasize to improve dietary diversity in selective food items. Further studies are needed to confirm this finding on other diet associated chronic diseases.

## Abbreviations

BMI: Body mass index; WC: Waist circumference; DDS: Diet diversity score; DDSP: Dietary diversity score with portions; FVS: Food variety score.

## Competing interests

The authors declare that they have no competing of interests.

## Authors’ contributions

RJ contributed to the data collection, data analysis and drafted the manuscript. NMB, MJS, PK and APH were the supervisory team on the project and contributed to the study design, data interpretation and revision of the manuscript. BY did the statistical analysis. All authors read and approved the final manuscript.

## Pre-publication history

The pre-publication history for this paper can be accessed here:

http://www.biomedcentral.com/1471-2458/13/314/prepub
